# Optimization of Ultrasound- and Microwave-Assisted Extraction for the Determination of Phenolic Compounds in Peach Byproducts Using Experimental Design and Liquid Chromatography–Tandem Mass Spectrometry

**DOI:** 10.3390/molecules28020518

**Published:** 2023-01-05

**Authors:** Thalia Tsiaka, Dimitra Z. Lantzouraki, Georgia Polychronaki, Georgios Sotiroudis, Eftichia Kritsi, Vassilia J. Sinanoglou, Despina P. Kalogianni, Panagiotis Zoumpoulakis

**Affiliations:** 1 Laboratory of Chemistry, Analysis & Design of Food Processes, Department of Food Science and Technology, University of West Attica, Ag. Spyridonos, 12243 Egaleo, Greece; 2 Institute of Chemical Biology, National Hellenic Research Foundation, 48, Vas. Constantinou Ave., 11635 Athens, Greece; 3 Analytical/Bioanalytical Chemistry & Nanotechnology Group, Department of Chemistry, University of Patras, 26504 Rio Patras, Greece

**Keywords:** peach byproducts, ultrasound-assisted extraction, microwave-assisted extraction, screening design, Box–Behnken design, phenolic compounds, liquid chromatography–tandem mass spectrometry, antioxidant activity

## Abstract

The conversion of plant byproducts, which are phenolic-rich substrates, to valuable co-products by implementing non-conventional extraction techniques is the need of the hour. In the current study, ultrasound- (UAE) and microwave-assisted extraction (MAE) were applied for the recovery of polyphenols from peach byproducts. Two-level screening and Box–Behnken design were adopted to optimize extraction efficiency in terms of total phenolic content (TPC). Methanol:water 4:1% *v/v* was the extraction solvent. The optimal conditions of UAE were 15 min, 8 s ON-5 s OFF, and 35 mL g^−1^, while MAE was maximized at 20 min, 58 °C, and 16 mL g^−1^. Regarding the extracts’ TPC and antioxidant activity, MAE emerged as the method of choice, whilst their antiradical activity was similar in both techniques. Furthermore, a liquid chromatography–tandem mass spectrometry (LC-MS/MS) method was developed and validated to determine chlorogenic acid and naringenin in byproducts’ extracts. 4-Chloro-4′-hydroxybenzophenone is proposed as a new internal standard in LC-MS/MS analysis in foods and byproducts. Chlorogenic acid was extracted in higher yields when UAE was used, while MAE favored the extraction of the flavonoid compound, naringenin. To conclude, non-conventional extraction could be considered as an efficient and fast alternative for the recovery of bioactive compounds from plant matrices.

## 1. Introduction

The globalization of trade, the drastic expansion of international exports concerning food commodities, and the increasing demand for the intensification of food production and supply chain are fostering the generation of enormous amounts of food and agro-industrial byproducts. According to the United Nations Food and Agriculture Organization (FAO), the residues produced from the initial processing of fruits and vegetables represent 20–40% of their total losses in their journey from the field to consumers’ tables. The biomass accumulated during agroindustrial processing shapes a major global environmental and socioeconomic issue, due to the emission of greenhouse gases, the pollution provoked by the disposal of material with organic content, and the consequent relegation of the quality of life in many developing countries. Thus, the management and re-valorization of food and agroindustrial byproducts, as part of sustainability and circular economies, constitute both one of the greatest challenges and also a unique opportunity for modern society [1,2,3].

Similarly to all plant matrices, peaches and their byproducts include a wide spectrum of secondary metabolites (i.e., polyphenols, sterols, terpenes, carotenoids, alkaloids, etc.) with important biological properties against metabolic syndromes, obesity, inflammation, cardiovascular diseases, oxidative stress, cancer, viruses, and bacterial activity, etc. [4,5]. Among peaches’ phytochemicals, phenolic compounds, such as phenolic acids, flavanols, flavonols, anthocyanins, etc., are bioactive molecules of particular interest for the food, nutraceutical, pharmaceutical, and cosmetic sectors, since they are related to the prevention and tackling of various pathological conditions, such as chronic non-communicable diseases [5]. On top of that, the peach cultivation and production, as well as peach canning, industry is one of the predominant agrifood activities in Greece [6]. As specified by Eurostat, Greece is one of the prime producers and exporters of nectarines and peaches in the European Union, with an annual production of more than 900,000 tons, which provide profits of nearly USD 380,000,000 [7]. In such a framework, the reuse and upcycling of peach byproducts, such as kernel, peel, leftover pulp, etc., through their conversion to value-added products renders a key strategy for the minimization and valorization of fruit byproducts [1]. In support of this, plant byproducts have been currently used as animal feeds, in edible films and coatings, in encapsulated functional products, as food additives, in supplements and nutraceuticals, etc. [8,9].

Focusing on the acquisition of high-quality extracts rich in bioactives, modern extraction technologies (i.e., pulsed electric field extraction, enzymatic extraction, ultrasound-assisted extraction, supercritical fluid extraction, microwave-assisted extraction, etc.) surface as promising alternatives to the more tedious conventional extraction methods (i.e., Soxhlet, maceration, percolation, solvent extraction, etc.). Their main advantages compared to traditional extraction are the high quality of the final extracts, the short extraction duration, the reduced volumes of organic solvents, their high selectivity and efficiency towards certain solutes, and their ability to use novel green extracting agents, such as natural deep eutectic solvent (NADES) [10]. Ultrasound-assisted (UAE) and microwave-assisted extraction (MAE), along with the supercritical fluid extraction (SFE), are the most established non-conventional extraction techniques. Acoustic cavitation produced by the compression and decompression of microbubbles and heating due to the ionic conduction and dipole rotation involve the mechanisms behind UAE and MAE, respectively, which facilitate the plant cell disruption, the increased solubility, and therefore the release of the compounds of interest in the solvent [11,12].

In contrast to classic extraction, several parameters play a definitive role on the extraction performance of non-conventional methods. Extraction solvent, extraction time, temperature, pressure, US or MW power, sonication duration, duty cycle, solvent/material ratio, and particle size are the most critical factors for the extraction efficiency [12,13]. Therefore, their optimization through the implementation of experimental design models (i.e., two-level designs, Plackett–Burman, central composite design, Box–Behnken design) in order to diminish the number of experiments and to assess the interactions between the extraction variables is of cumulative importance [14].

Taking into consideration the current advances and trends in the market of plant byproducts, the main objective of the present work was the optimization of UAE and MAE in order to obtain phenolic-rich extracts with high antioxidant and antiradical activity. To go a step further, an LC-MS/MS method was developed and validated in order to determine the effect of UAE and MAE on the content of two characteristic phenolic compounds of different classes, chlorogenic acid and naringenin. To our knowledge, 4-chloro-4′-hydroxybenzophenone was used for the first time as internal standard for LC-MS/MS analysis of foods and natural products. To conclude, the overall aim of this study was to suggest efficient and eco-friendlier methodologies for peach byproducts future valorization.

## 2. Results and Discussion

### 2.1. Optimization of UAE and MAE by Implementing Design of Experiments (DOE) Models

Extraction time (min, X_1_), sonication duration of pulse sequence at ON mode, for UAE (s) or extraction temperature, for MAE (°C), and solvent/material ratio (mL g^−1^, X_3_) were the investigated extraction factors or independent variables. Total phenolic content (TPC) was used as the dependent variable or response in both 2^3^ full-factorial and Box–Behnken designs. Methanol–water 4:1 *v*/*v* mixture was selected as the extraction solvent for both extraction techniques since alcohol–water binary solvent systems enhance the polyphenols extraction due to the increased affinity to the target compounds [15]. Furthermore, solvents with high polarity and surface tension and relatively low viscosity and vapor pressure (such as water and methanol) facilitate cell disruption and release of the extracted compounds, since they enhance the cavitation intensity of ultrasounds (US) and increase cell permeability [16,17]. Polar solvents are also ideal for MAE because -OH bonds enable microwave (MW) energy absorption, and as consequence enable the increase in temperature in the extraction system that promotes the release of the compounds of interest into the solvent [15]. According to recently published studies [18,19,20], higher values of US power implemented at relatively short extraction times result in higher extraction yields of bioactive compounds. Thus, US power was set at 600 W and extraction temperature was kept constant at 35 °C by immersing the extraction vessel into an ice bath. Moreover, MW energy was adjusted at 50 W in order to provide energy to the extraction system, which will provoke a gradual, not abrupt, increase in the extraction temperature to reach the values defined in the DOE models.

#### 2.1.1. Screening Design (2^3^ Full Factorial Design)

The initial step of all optimization processes requires the screening of the parameters under study and of their values’ range. Therefore, the 2^3^ full factorial screening design is essential to navigate the response surface methodology (RSM) Box–Behnken design to a more constricted value range of the extraction factors, where the total phenolic content (TPC) of peach byproducts extracts appears to be optimized. The experimental runs of both screening and RSM models were randomized to avoid any systematic errors in the final results. The results of 2^3^ design are demonstrated in Table S1.

The determination coefficient (R^2^) and the determination coefficient adjusted for the degrees of freedom (R^2^_adj_) show the prediction ability and suitability of the constructed DOE models. In order for a model to validly predict the experimental response (i.e., TPC) under certain values of independent variables (i.e., extraction factors), the values of R^2^ and R^2^_adj_ should be higher than 0.8 and the difference between them should be roughly equal to 0.2. Regarding the two proposed screening models, one for each extraction technique, both are considered reliable enough (R^2^ = 0.79 and R^2^_adj_ = 0.50 for UAE and R^2^ = 0.90 and R^2^_adj_ = 0.76 for MAE) to direct the subsequent Box–Behnken designs to the optimal conditions. 

The linear and interaction terms of the extraction factors with very high p-values (over 0.35) were considered as nonsignificant terms and were omitted from the final 2^3^ designs. The screening design for MAE indicated that special focus should be given to select the value range of all three factors while constructing the BBD model, since the interaction between extraction time and pulse sequence ON, x_1_x_2_ (*p*-value = 0.04) followed by the interaction of extraction time and solvent/material ratio, x_1_x_3_ (*p*-value = 0.09) seemed to play an important role on the TPC of the peach extracts (Table S2). On the other hand, in the case of UAE, the phenolic content of peach byproducts appeared to be affected most by the values of solvent/material ratio, x_3_ (*p*-value = 0.09) (Table S2). The two-dimensional (2D) contour plots pinpointed that UAE would be optimized at higher extraction times, lower sonication duration, and low-to-medium solvent-to-material ratios, whilst TPC of MAE extracts would be increased at longer extraction durations, increased temperatures, and lower-to-medium solvent/material ratios (Figure S1a–f).

#### 2.1.2. Response Surface Methodology (RSM) for the Optimization of UAE and MAE: Box–Behnken Designs

The extraction processes were optimized by applying two symmetrical 16-run Box–Behnken (BBD) designs, which were directed by the results of the aforementioned screening designs. Based on the 2^3^ designs, extraction time should be studied at values between 15–25 min in both techniques, while higher TPC values seems to be achieved at a solvent/material ratio between 15–35 and 15–25 mL g^−1^ in UAE and MAE, respectively (Figure S1a–f). In regard to the sonication duration, the values between 10 to 20 s ON appears to maximize the phenolic content of the extracts, while the extraction temperatures of interest in MAE were determined at the region between 60–70 °C (Figure S1a–f). The results of the BBD models are presented in Table S1. 

Based on the experimental data of the BBD models the measured response (i.e., TPC of peach dry byproducts) for each technique is described by a second-order polynomial equation (Equations (1) and (2)). The equations terms with high p-values were excluded from the models as nonsignificant. In all cases, the negative signs in some of the terms of Equations (1) and (2) imply the decrease in TPC of peach pulp extracts when the value of these specific terms is increased.

UAE TPC (mg of GAE g^−1^ dry sample) = 1.9203 + 0.00019 x_1_^2^ − 0.036 x_2_ + 0.0020 x_2_^2^ − 0.0024 x_3_ + 0.00070 x_3_^2^ − 0.000002 x_1_x_2_^2^ − 0.00032 x_1_x_3_ − 0.0014 x_2_x_3_ (1)

MAE TPC (mg of GAE g^−1^ dry sample) = 13.1891 + 3.4485 x_1_ − 0.1228 x_1_^2^ − 1.1334 x_2_ + 0.0102 x_2_^2^ + 0.4656 x_3_ − 0.0036 x_1_x_2_ − 0.00027 x_1_x_2_^2^ + 0.0011 x_1_^2^x_2_ − 0.0908 x_1_x_3_ + 0.0023 x_1_^2^x_3_ + 0.0058 x_2_x_3_ (2)

**Figure 1:**
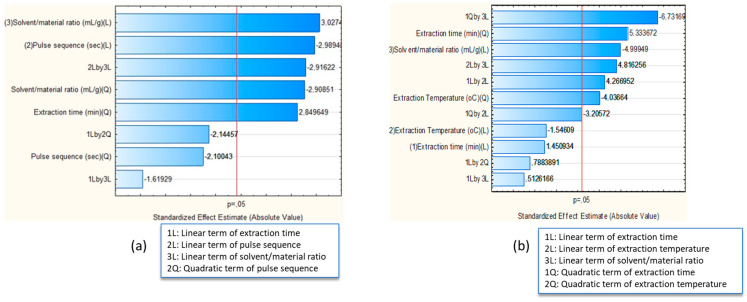
Pareto charts of BBD models for (**a**) UAE and (**b**) MAE.

The terms of the two equations were recorded, according to their significance, in Pareto charts (Figure 1a,b), where the terms which were critical for the extraction of phenolics from peach byproducts exceeded the threshold of *p*-value ≤ 0.05, depicted by the red line.

Based on the Pareto chart (Figure 1a) and the ANOVA table (Table S3), the most crucial parameters for UAE optimization were (a) the quadratic term of extraction time (x_1_^2^), (b) the linear term of pulse sequence ON (x_2_), (c) the linear (x_3_^2^) and quadratic terms (x_3_) of solvent/material ratio, and (d) the interaction of the linear term of pulse sequence and the linear term of solvent/material ratio (x_2_x_3_). In a similar vein, the Pareto chart (Figure 1b) and ANOVA table (Table S3) of MAE revealed as key factors for the extraction of phenolics (a) the quadratic term of extraction time (x_1_^2^), (b) the quadratic term of extraction temperature (x_2_^2^), (c) the linear term of solvent/material ratio (x_3_), (d) the interaction of the linear term of temperature with the linear (x_1_x_2_) and quadratic term (x_1_^2^x_2_) of extraction time, and (e) the interaction of the linear term of solvent/material ratio to the quadratic term of extraction time (x_1_^2^x_3_) and to the linear term of temperature (x_2_x_3_). 

The R^2^ and R^2^_adj_ values (R^2^ = 0.887 and R^2^_adj_ = 0.757 for UAE and R^2^ = 0.986 and R^2^_adj_ = 0.946 for MAE) of BBD designs for both extraction methods proved the models were reliable and valid. As indicated by the R^2^ values, almost 90% and 99% of the total variations of UAE and MAE, respectively, was explicated by the generated BBD models. Additionally, the low values of MS_residuals_ of the two BBD models (Table S3) demonstrated that the predictability of the models was high since no significant differences between the actual (measured) and estimated TPC values were expected. The four replicates in the center points (0, 0, 0) were used to evaluate the robustness of the BBD models and of the developed experimental protocol. The low values of standard deviation of the four central experimental runs (stdev = 0.056 and stdev = 0.060, for UAE and MAE, respectively) confirmed that the extraction processes were repeatable.

#### 2.1.3. Effect of UAE and MAE Extraction Factors on TPC of Peach Byproducts

**Figure 2:**
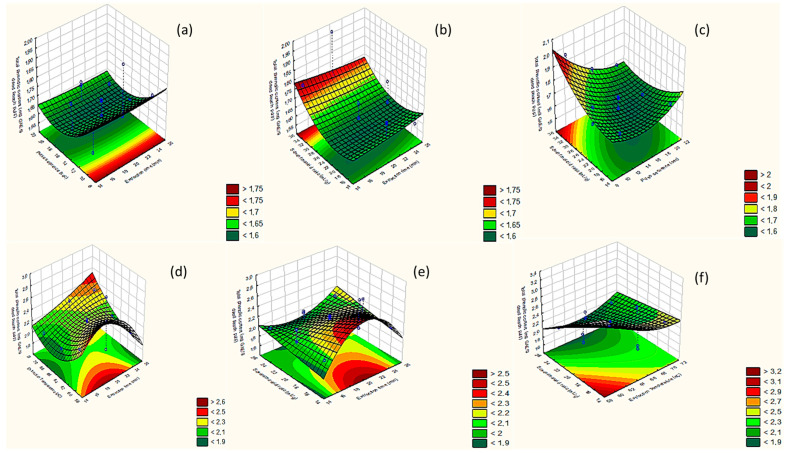
RSM plots of (**a**) extraction time vs. pulse sequence ON; (**b**) extraction time vs. solvent/material ratio; (**c**) pulse sequence vs. solvent/material ratio; (**d**) extraction time vs. extraction temperature; (**e**) extraction time vs. solvent/material ratio; (**f**) extraction temperature vs. solvent/material ratio.

The three-dimensional (3D) response surface plots were used to assess the effect of extraction factors on the TPC values of peach pulp extracts (Figure 2a–f). The 3D plots portray the interaction of two, each time, of the studied extraction parameters, while the third parameter is always kept at the medium value level (0).

##### Extraction Time

As presented in Figure 2a,b and in Figure 2d,e, the TPC content was increased when extraction times from 15 to 20 min were applied, regardless of the extraction technique that was implemented. In line with the present findings, Foudjar et al. (2019) reported that the gradual increase in extraction time from 10 to 15 min enhanced the TPC values due to the creation of US microbubbles which promote cell disruption and the release of phenolic compounds in the extracting agent. Nonetheless, a more extended duration of UAE may be conducive to the degradation or oxidation of polyphenols [21]. Concerning MAE, a higher MW power significantly reduced the extraction time (from seconds to less than 5 min) [22,23]. In the current work, a relatively low MW power (i.e., 50 W) was applied; therefore, a 20 min extraction was required to obtain high TPC values.

##### US Pulse Sequence ON Mode

As shown in Figure 2b,c, the extraction efficiency was maximized when the sonication duration was around 8–10 s. In relation to the current literature, a sonication period of 8 to 10 s ON and 5 s OFF was suitable to avoid the degradation of more susceptible phenolic constituents, such as phenolic acids. An increase in the ultrasonic treatment or even a continuous sonication amplifies the sonochemical and cavitation effects, resulting in the production of more free hydroxyl radicals, which compromise the stability of phenolic compounds [24].

##### Extraction Temperature of MAE

In agreement with the results of Alara et al. (2019), the increase in MAE extraction temperature from 40 to 60 °C raised the rate and diffusion of the extracted compounds (Figure 2d,f). However, a further increase in temperature may present a negative impact on the final TPC due to the reduction and deterioration of both solvents and targeted bioactives [25].

##### Solvent/Material Ratio

In general, the use of higher solvent volumes during the extraction process enables the mass transfer to the solvent system and, therefore, the solubility of the compounds of interest. Nonetheless, a constant increase at the volumes of the extracting solvent does not always lead to higher extraction yields due to the equilibrium that is restored, after a critical point, in the mass transfer between the plant matrix and the solvent [26,27].

According to the outcomes of our study, UAE for the recovery of phenolic compounds from peach byproducts was optimized at solvent/material ratios within the limits of 30–35 mL g^−1^ (Figure 2b,c), while the higher phenolic yield for MAE was achieved at the region of 15–20 mL g^−1^ (Figure 2e,f).

#### 2.1.4. Optimal Conditions of UAE and MAE

The assessment of RSM plots suggested three experiments at specific values of the extraction parameters where the TPC reached the higher values in order to define the exact optimal conditions of UAE and MAE (Table S4). The experimental and predicted, by Equations (1) and (2), TPC values were close, hence the BBD models of UAE and MAE showed good predictability regarding the phenolic content of peach byproduct extracts. The final optimal values of the extraction factors are presented in Table 1.

According to the results of the Folin–Ciocalteu method for the determination of phenolic content of UAE and MAE peach extracts, MAE turned up as a more efficient extraction approach (*p*-value of TPC of extracts at optimal conditions ≤ 0.05) than UAE. The difference in the phenolic yield of the two non-conventional extraction methods could be attributed to the higher temperatures in combination with the longer extraction duration of MAE. The higher MAE temperature reduces the viscosity of the used solvents and their surface tension, which intensify the diffusion of the extracted compounds and therefore the extraction rate [28]. Furthermore, the increased temperatures enable the release of phenolics from their esterified and glycoside forms, contributing in this way to the higher final polyphenols content of MAE [29,30]. On the other hand, the sonication of polar solvent systems, such as methanol–water, may result in the generation of free radicals, which could cause the oxidation and degradation of the phenolics [24].

### 2.2. Antiradical and Antioxidant Activity

The optimal high energy extracts bearing the highest total poly(phenolic) content (TPC) among DOE-tested extracts as depicted from the Folin–Ciocalteu assay were appointed for further investigation on their antioxidant potential. Table 2 demonstrates the results for the radical scavenging activity on ABTS^●+^, and the reducing/antioxidant power as evaluated by the FRAP method for UAE and MAE optimal extracts.

Apparently, the optimal MAE extraction was proven slightly more efficient than the optimal UAE extraction in recovering antioxidant compounds with reducing capacity towards Fe(III) (*p <* 0.05). The results agree with TPCs (Table 1), which denoted MAE extract as richer in phenolic constituents than UAE extract (*p <* 0.05), signifying that the higher the (poly)phenolic concentration, the higher the reducing/antioxidant activity observed for the extracts under study. Further, according to Table 2, the antiradical content of the extracts corresponding to mg Trolox equivalents (TE) per gram of dry peach byproduct was comparable in the studied extracts (*p* > 0.05). It may be assumed that more chemical species, other than the (poly)phenolic compounds depicted in the TPC and FRAP results, may be involved in the binding of the free radical for ABTS^●+^ assay. Such compounds, such as vitamins C and E [31,32,33], and amino acids or proteins [33,34,35], typically found in peach and its products, were proportionally extracted in the UAE and MAE extracts. Alternatively, synergistic effects occurring between antiradical antioxidants in both extracts may have leveled the results of scavenging activities [36,37]. Nonetheless, it is reported that the antiradical potential of natural products does not necessarily match the reduction of Fe(III) to Fe(II) as measured by FRAP [38].

It is also noteworthy that the measurement upon the time plateau (T_plateau_) leads to almost arithmetically equal results; 9.84 (±0.95) and 9.83 (±0.95) mg TE·g^−1^, for UAE and MAE, respectively (Table 2). Specifically, although both extracts showed statistically same results (*p* > 0.05) at either 5 min or 3 h (T_plateau_) absorbance monitoring, a stronger percentage difference of 6.3% was observed between samples at 5 min, compared with 3 h where the difference was merely 0.1%. As indicated by several authors [38,39,40], the rate of a reaction involving a free radical, such as ABTS^●+^ and 2,2-diphenyl-1-picrylhydrazyl (DPPH^●^), varies widely among phenolic substrates and solution concentrations, from minutes to hours. However, the simplification of the procedure is popularized by setting the wrongfully assumed reaction time and the corresponding measurement at 5 or 30 min. It is thus crucial to consider not only the antiradical content but also the reaction time of the scavenging reaction until equilibrium plateau. This finding highlights the importance of following appropriate chemical practices to obtain true and highly reproducible results.

### 2.3. LC-MS/MS Determination of Targeted Phenolic Compounds

Further investigating the phytochemical potential of the studied UAE and MAE extracts, a signature antioxidant phenolic compound that predominates peach pulp and byproducts, namely chlorogenic acid [32,41,42], and naringenin as a representative flavonoid [43,44], were determined with a targeted HPLC-ESI-QqQ-MS/MS(MRM) analysis. Furthermore, naringenin is a flavonoid with bioactive properties rendering it a potent biomolecule for application in food or pharmaceutical preparations; nonetheless, its intrinsic astringency may limit its usage as a product ingredient, especially in high concentrations [45,46]. It is thus important to determine naringenin content in natural products that are intended for incorporation in foodstuffs or orally administrated drugs, such as the peach byproduct examined in our work. Moreover, we introduced the chlorinated phenolic compound 4-Chloro-4’-hydroxybenzophenone (CAS No 42019-78-3) as a suitable and effective internal standard (IS) to improve the precision and accuracy of quantitation.

4-chloro-4’-hydroxybenzophenone may be found in a synthetic formulation used as a pharmaceutical intermediate [47]. Moreover, the standard compound can be purchased at a relatively low cost and high purity, and it is feasibly dissolved in common solvents, such as methanol and ethanol. Additionally, it is structurally related to phenolic compounds, either natural or synthetic, as it possesses a distinct phenolic structure. Most importantly, 4-chloro-4’-hydroxybenzophenone has not been yet identified in any natural source to our knowledge, thus it stands as a perfect fit for IS in determination of (poly)phenolic compounds in foods, byproducts, and natural products [48,49]. The only drawback observed in the scope of method validation (see Supplementary Materials) was its instability when added to quality control samples for one month period at −20 °C. However, the phenomenon was attenuated by preparing fresh working solutions of the compound prior to the addition to samples and calibrators.

For mass spectrometric investigations, a precursor deprotonated ion [M-H]^–^ and two MRM transitions presenting the better signal response have been established for each molecule. The two predominant fragments were set as quantifier (QF) and qualifier (QL) ions for the method acquisition in the product ion scan mode.

**Figure 3:**
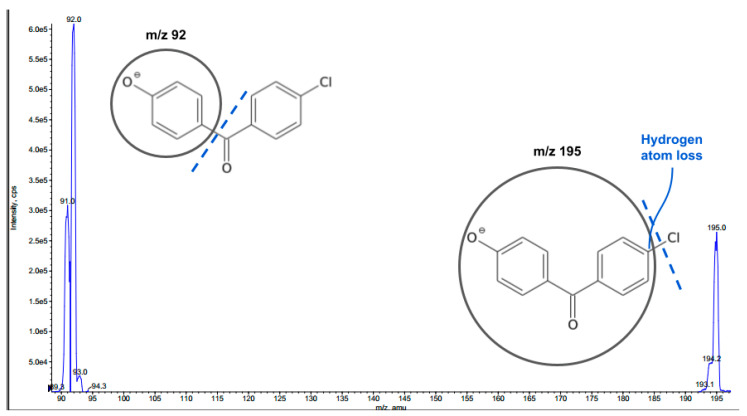
Mass spectrum of 4-Chloro-4’-hydroxybenzophenone (IS) acquired under electrospray negative ionization (ESI-) and product ion scan (MS^2^) of precursor ion, and proposed MS^2^ fragmentations.

Internal standard formed [M-H]^–^ ion (*m/z* 231.0) when subjected to ESI in negative ion mode. Figure 3 illustrates the product ions with measurable signal intensity observed in MS^2^ spectrum under the conditions set for our method, and possible fragmentations proposed for the precursor ion. The most abundant product ion at *m/z* 92, which arose from loss of C_7_H_4_ClO chemical moiety, was chosen to set up multiple reaction monitoring (MRM) transitions used for quantitation of IS. MRM transitions of the second most intense product ion 231.0 > 195.0 (*m/z*) were also recorded for further structure identification (qualifier ion).

Concerning targeted phenolic compounds, the following fragmentation of MRM transitions (*m/z*) of deprotonated precursor to daughter ions were proposed:

Chlorogenic acid: (i) 353.0 > 191.10 (QF), where the product ion corresponds to [M-H]^–^—caffeoyl; (ii) 353.0 > 84.8 (QL), where diagnostic *m/z* 84.8 is presumably assigned to crotonate ion (C_4_H_5_O_2_^–^).

Naringenin: (i) 270.9 > 151.0 (QL), occurring from [M-H]^–^ (A-ring fragment); (ii) 270.9 > 119.10 (QF) corresponding to [M-H]^–^ (B-ring fragment).

**Figure 4:**
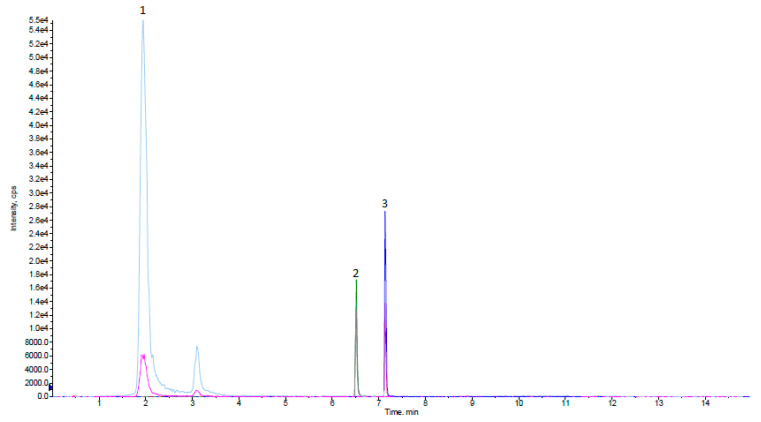
Chromatographic/MS^2^ (MRM) peaks of standard compounds: (1) chlorogenic acid, (2) naringenin, and (3) internal standard in methanol-0.1% (*v/v*) aqueous formic acid solution.

Identification of analytes and IS in the studied samples and standard solutions was based on the comparison of their retention times and on the relative abundance of the MS^2^ product ion signals. As shown in Figure 4, all standards demonstrated adequate chromatographic separations with characteristic retention times at 1.9 min for chlorogenic acid, 6.5 min for naringenin, and 7.2 min for IS.

**Figure 5:**
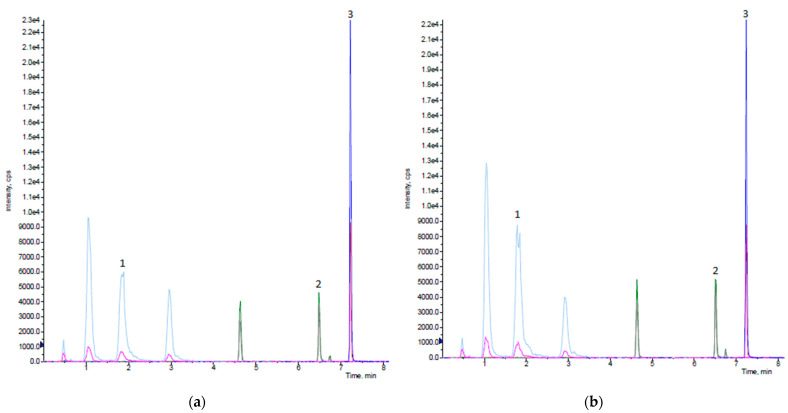
HPLC-MS/MS(MRM) chromatograms of standard phenolic compounds (1) chlorogenic acid, (2) naringenin, and (3) internal standard in: (**a**) UAE extract; (**b**) MAE extract.

Furthermore, the absence of endogenous interfering or co-eluting peaks at the retention times and with specified MRM transitions of analytes and IS, when spiked in peach byproduct matrix, was considered as evidence for the selectivity of the method. The background noise was also minimized upon the optimized MS conditions, offering high precision and low minimum detection and quantitation limits (see Supplementary Materials). Representative MS/MS(MRM) chromatograms of UAE and MAE extracts are illustrated in Figure 5a,b, respectively.

Quantitation of target phenolic compounds was conducted in each stage of the experimental design, for the extracts that presented the higher and lower yield of total (poly)phenolic content as evaluated with the Folin–Ciocalteu assay (Table 3). The specific extraction conditions, as well as TPCs, for each case of selected UAE and MAE methods are presented in Table 1 and Table S1 and according to Table 4.

As observed in Table 3 for 2^3^ design runs, UAE samples extracted for 25 min with ultrasonic pulse sequence at 10 s recovered similar amounts of chlorogenic acid and naringenin (*p >* 0.05). MAE/run 7 with a longer extraction time of 25 min presented the highest performance in TPC and target phenolic compounds recovery (*p <* 0.05), among 2^3^ design experiments (Table 3 and Table S1).

In the case of BBD design-derived runs, UAE/run 11 extraction conditions of a prolonged 20 min extraction period, shorter sonication pulse sequence at 10 s and a high solvent–dry material ratio of 35:1 (*v/w*) resulted in extracts significantly richer in TPC (Table S1), as well as chlorogenic acid and naringenin (*p <* 0.05) (Table 3). According to MAE results from the BBD study, longer extraction time at 20 min, lower temperature at 60 °C and lower solvent–dry material ratio of 15:1 (*v/w*) proved more efficient for acquiring the highest TPC among all BBD extracts for both high energy extractions (Table S1), as depicted in the results for MAE/run 9. Notwithstanding, the specific extraction conditions were not proportionally effective in retrieving the targeted phenolics compared with the extracts that provided the higher TPC among BBD experiments (Table 3). Since the optimization of the extraction parameters was carried out based on the potential for enhancing the total (poly)phenolic concentration of the prepared extracts, it is possible that the optimized conditions also favor the recovery of other (poly)phenolic compounds found in peach fruit, peel, and byproducts, such as neochlorogenic acid, protocatechuic, *p*-hydroxybenzoic, *p*-hydroxyphenylacetic, chlorogenic, *p*-coumaric, and ferulic acids, quercetin glycosides, kaempferol-3-O-glucoside, catechin, and cyanidin-3-glucoside, over chlorogenic acid and naringenin [50,51].

Furthermore, the optimal UAE (15 min extraction time; 8 s ON-5 s OFF sonication pulse; 35 mL·g^−1^; 600 W; 35 °C), and MAE (20 min extraction time; 16 mL·g^−1^; 50 W; 58 °C) were examined for their content in target compounds (Table 3). Remarkably, according to our results, chlorogenic acid was recovered in a higher degree with UAE optimal extraction (*p <* 0.05), while optimal MAE proved the most efficient in extracting naringenin among all studied extraction conditions (*p <* 0.05).

## 3. Materials and Methods

### 3.1. Chemicals and Standards

Folin–Ciocalteu’s phenol reagent, ferric chloride hexahydrate (FeCl_3_·6H_2_O), ferrous sulfate heptahydrate (FeSO_4_·7H_2_O), and hydrogen chloride (hydrochloric acid ≥ 37% *w/w*) were purchased from Chem-Lab NV (Zedelgem, Belgium). Sodium carbonate was obtained from Carlo Erba Reagents (Cornaredo, Italy), and absolute ethanol and acetic acid from Merck KgaA (Darmstadt, Germany). Gallic acid (3,4,5-trihydroxybenzoic acid), 2,2′-azino-bis(3-ethylbenzothiazoline-6-sulfonic acid ammonium salt) (ABTS non radical form) and 2,4,6-tris(2-pyridyl)-s-triazine (TPTZ) were bought from Alfa Aesar (Karlsruhe, Germany), while Trolox (6-hydroxy-2,5,7,8-tetramethylchroman-2-carboxylic acid) and potassium persulfate were supplied from Sigma-Aldrich (St. Louis, MO, USA). Standard phenolic compound of chlorogenic acid (≥99%) was purchased from Extrasynthese (Genay, France), and naringenin (97%) and 4-chloro-4’-hydroxybenzophenone (97%) were from Alfa Aesar (Karlsruhe, Germany). Formic acid was bought from LGC Standards (Wesel, Germany). All reagents used for the spectrophotometric assays were of analytical grade (≥94%). Solvents used for all experimental analyses were of LC-MS grade and were purchased from Sigma Aldrich Co. (Gillingham, United Kingdom), Carlo Erba Reagents (Val de Reuil, France), Chem-Lab NV (Zedelgem, Belgium), Fischer-Scientific (Loughborough, UK), and Merck KGaA (Darmstadt, Germany).

### 3.2. Peaches Byproducts and Sample Preparation

Peach (*Prunus persica* L.) pulp was kindly provided by Danais S.A. Fruit Processing Industry & Export Company (www.danais-sa.com, accessed on 4 January 2023) (Skydra, Pella, Greece). Peaches byproducts (i.e., peach pulp) were produced during the processing and compression of raw fruits with a particle size of over 0.5 mm, which did not meet the criteria for sale or for producing compotes and preserves. They mainly consisted of the peach skin and flesh. Peach pulp was derived from the fruits of «Caterina», «Loadel», «Fortuna», «A-37», «Andross», and «Everts» clingstone varieties, which were collected from the regions of Pella and Imathia, Central Macedonia, Greece, from the beginning of July until the end of September. 

Peach pulp was lyophilized, and dry material was homogenized and powdered in a laboratory mill (Type ZM1, Retsch GmbH, Haan, Germany). The moisture of the pulp samples was determined at 90.37 (±0.510) % (*w/w*). All samples were kept in glass bottles in −20 °C in darkness until further treatment. 

### 3.3. Ultrasound-Assisted Extraction (UAE) and Microwave-Assisted Extraction (MAE)

The ultrasound-assisted extraction (UAE) was conducted by a Vibra-Cell VCX 750 (20 kHz, 750 W) ultrasonics processor (Sonics and Materials Inc., Newtown, CT, USA), equipped with a piezoelectric converter and 13 mm diameter probe fabricated from titanium alloy Ti–6Al–4V. Microwave-assisted extraction (MAE) was performed using a CEM Focused Microwave Synthesis System, Model Discover (CEM Corporation, Matthews, NC, USA) in open-vessel mode with a reflux system installed over the open cell.

In both techniques, one gram of dried pulp and different volumes of a methanol:water 4:1 *v*/*v* mixture were used for the extraction of phenolic compounds. Extraction time, sonication duration (for UAE) or extraction temperature (for MAE), and solvent/material ratio were the parameters under optimization. In UAE, ultrasound power and sonication intervals were set at 600 W and 5 s, respectively, in all experiments, while extraction temperature was also kept constant at 35 °C by placing the extraction vessel into an ice bath. In MAE experiments, microwave power was set at 50 W. 

After extraction, the extracts were filtered under vacuum and aliquots of the supernatants were subjected to dryness using a speed vac concentrator. Then, the dry residues were stored at −20 °C to be used for the spectrophotometric and LC-MS/MS analyses.

### 3.4. Experimental Design (DOE) Models

A two-level full factorial design, 2^3^, and a symmetrical 16-run three-level Box–Behnken design (BBD) were applied for the screening and the optimization of the extraction factors, respectively. The extraction factors under optimization were the (a) extraction time, X_1_ (min); (b) sonication duration as pulse sequence ON mode (s), for UAE, or extraction temperature (°C), for MAE, X_2_; and (c) solvent/material ratio, X_3_ (mL g^−1^). The experimental response of DOE models was the total phenolic content of the extracts, expressed as mg of gallic acid equivalents (GAE) per gram of dry peach pulp.

Since the real values (X_1,_ X_2,_ X_3_) of the extraction factors are expressed in different physical units (i.e., minutes, Celsius degrees, seconds, volume-to-weight), they were switched and normalized to coded dimensionless values (x_1_, x_2_, x_3_) to provide unbiased and reliable results [52]. The real and normalized values of the extraction parameters for the DOE models of the two extraction techniques are presented in Table 4.

### 3.5. Determination of Total Phenolic Content

The total (poly)phenolic content (TPC) of each extract was evaluated applying a Folin–Ciocalteu colorimetric micromethod based on the methodology by Lantzouraki et al. (2015) [53] and using the UV-vis spectrophotometer Spectro 23RS (LaboMed, Inc., Los Angeles, CA, USA). The final results were expressed as micrograms gallic acid equivalents (GAE) per gram dry peach pulp using a calibration curve, where concentrations of standard solutions ranged from 20 to 250 mg·mL^−1^ gallic acid (Y = 0.0035X − 0.081, R^2^ = 0.999). All determinations for tested samples were carried out at least in triplicate.

### 3.6. Antiradical Activity against ABTS^●+^ Radical

The antiradical activity of the extract solutions was determined as described in a previous work [53]. The ABTS^●+^ assay provides an estimate of the potency of samples to scavenge ABTS^●+^ free radicals, which was expressed as the concentration of Trolox equivalents (TE) per gram dry peach pulp using a respective standard curve (concentration range: 0.10–4.0 mM, Y = 0.25X + 0.018, R^2^ = 0.998). The absorbance was recorded at 734 nm for two time points, i.e., at 5 min upon the initiation of the chemical reaction, and at plateau of time (T_plateau_) where absorbance stabilizes at a minimum value (color stabilization) signifying the end point of the reaction. For the tested sample solutions, T_plateau_ was reached at approximately 3 h. All measurements were conducted in triplicate.

### 3.7. Ferric Reducing/Antioxidant Power (FRAP) Assay

The antioxidant capacity of the extracts was determined based on the reduction of iron from the ferric to the ferrous form when complexed with 2,4,6-tris(2-pyridyl)-s-triazine. The FRAP assay was carried out according to our previous work [54]. Standard aqueous solutions of ferrous sulfate heptahydrate with ranging concentration from 10 to 4000 μM were prepared for the construction of the standard curve (Y = 0.25X + 0.018, R^2^ = 0.998). The absorbance at 595 nm was measured every 60 sec until stabilization to a maximum value, observed at approximately one hour (T_plateau_). The results were expressed as mg Fe(II)·g^−1^ dry peach pulp. Measurement for each sample or standard was repeated three times.

### 3.8. LC-MS/MS Quantitative Analysis of Targeted Phenolic Compounds

Chlorogenic acid and naringenin were determined with a targeted HPLC-ESI-QqQ-MS/MS(MRM) analysis. To this end, a rapid, sensitive, and robust methodology was developed and validated. A paramount aspect for the LC–MS method development and validation was the fusion of the two extract types, i.e., UAE and MAE, to achieve a combined chemical composition for the coequal contribution of each extract into the stipulation of chromatographic and MS conditions. 4-Chloro-4’-hydroxybenzophenone (CAS No 42019-78-3) was used as the internal standard (IS).

#### 3.8.1. Preparation of Samples and Standards

The delivered UAE and MAE extracts were lyophilized, and the dry residues were subsequently redissolved in a 1:1 (*v/v*) mixture of methanol and 0.1% (*v/v*) aqueous formic acid solution to a final concentration of 15 mg dry matter·mL^−1^. For the combined solution of dry extracts, the final concentration for each extract type was 7.5 mg dry matter·mL^−1^. The solutions for analysis were filtrated through PTFE syringe filters (13 mm diameter, pore size 0.45 μm pore size) before being injected into the LC port. Moreover, 1000 μg·mL^−1^ stock solutions were prepared in methanol for each phenolic standard and for IS, which were further diluted with methanol–water (0.1% *v/v* formic acid) at a ratio 1:1 (*v/v*) to varying final concentrations as required by the experiments, for pre- and post-spiked studies and quality controls as well. Internal standard concentration in all solutions was 1.0 μg·mL^−1^. The sample and standard solutions were stored at −20 °C in darkness until further analysis.

#### 3.8.2. HPLC Method

The liquid chromatographic analysis was conducted on an Agilent 1200 HPLC system equipped with a binary pump, a thermostated column compartment, a micro vacuum degasser, and an autosampler (Agilent, Santa Clara, CA, USA). The chromatographic separation of compounds was achieved with a reversed-phase Zorbax Eclipse Plus C-18 column (50 mm length, 2.1 mm inner diameter, 3.5 μm particle size; Agilent, Santa Clara, CA, USA) fitted with an Agilent RRLC column in-line filter (2.1 mm inner diameter, 0.2 μm pore size). The column was maintained at room temperature (approximately 20 °C), and a 2 μL aliquot from samples, quality controls, standard solutions, or blanks was injected for each run. The developed method was based on the elution conditions of Tsiaka et al. (2022), which were modified accordingly [55]. In detail, the mobile phase used for separations consisted of 0.2% (*v/v*) formic acid in water (eluent A) and 0.1% (*v/v*) formic acid in acetonitrile (eluent B). The gradient elution program initiated at a flow rate of 300 μL·min^−1^ with 10% B, followed by a linear gradient to 20% B in 0.50 min, and to 30% B at 4.0 min. At 4.1 min, the flow rate increased to 350 μL·min^−1^ and the gradient changed linearly from 30% to 50% B, then held constant for 0.40 min, turned to linear gradient up to 65% B at 5.1 min, linear 65–100% B at 7.0 min, purged with 100% B for 1.0 min, then held steady at 100% B for one more minute with a flow rate of 300 μL·min^−1^, and a linear gradient back to 10% B at 9.1 min. Column re-equilibration lasted for 5.9 min with 10% B, giving a total run time of 15 min. Furthermore, blank solvent samples ran between injections, and an injection-valve cleaning program was also used prior to injections and at the end of the equilibration period to diminish the possible carry-over effect from the previous analysis. All measurements were conducted at least in triplicate. The retention times for each compound are presented in Table 5.

#### 3.8.3. Mass Spectrometric Analysis and Data Interpretation

A 3200 Q TRAP triple-quadrupole linear ion trap mass spectrometer coupled with the HPLC system and interfaced with a Turbo VTM source and a Turbo Ion Spray probe (SCIEX, Framingham, MA, USA) was used for the mass spectrometric analyses. An electrospray ionization source was operated for ionizing the molecules in negative mode (ESI-). Trials were performed in both positive and negative ionization mode, and it was revealed that the negative ionization provided a better signal response over the positive mode, after direct infusion under different MS conditions. A series of experiments were performed to select the optimized analyte-specific MS settings for each compound. The parameters of the ionization source were further improved with flow injection analysis (FIA). General ion source settings included curtain gas (10 psi), ion spray needle voltage (−4.500 V), temperature (600 °C), nebulizer gas (50 psi), heater gas (50 psi), interface heater (ON set), and collisionally activated dissociation gas (nitrogen gas; medium mode). Multiple reaction monitoring (MRM) ion transitions and the respective parameters were investigated by injecting standard solutions at a concentration of 2.9 μg·mL^−1^ using a syringe pump for a continuous flow. A precursor molecular ion and two main MS^2^(MRM) transitions have been selected for each compound. The MRM parameters were further optimized with a ramp scheme to acquire higher sensitivity (Table 5). The Analyst Software program (version 1.4.2; SCIEX, Framingham, MA, USA) was employed for data acquisition, evaluation, processing, and instrument control.

#### 3.8.4. Method Validation

The validation of the method was conducted according to official guidelines [48,56,57] considering the following parameters: selectivity, linearity, limits of detection (LOD) and quantitation (LOQ), accuracy, precision, matrix effect, total (process) recovery, as well as stability of standard compounds [44,58,59,60]. Detailed data are presented in the Supplementary Material (Tables S5–S9).

### 3.9. Data Analysis

Experimental design models, data, and graphs were generated by the Statistica package (Version 12, trial version, TIBCO Software Inc., Palo Alto, CA, USA). All measurements were realized at 95% (*p*-values ≤ 0.05) confidence level. The final results for the spectrophotometric determinations and LC-MS/MS quantification of the targeted phenolic compounds in peach extracts were reported as means along with the standard deviations per sample. Values were evaluated by one-way analysis of variance (ANOVA), followed by Tukey’s honest significance test. P values lower than 0.05 (*p* < 0.05) were statistically significant. All statistical calculations, including partial correlations and curve fittings, were performed with the OriginPro 8 SR0 software (OriginLab, Northampton, MA, USA).

## 4. Conclusions

Nowadays, the efficient use of plant byproducts is essential to pave the way for their re-introduction as novel products of potential commercial value. For this scope, non-conventional high energy extraction techniques are now widely employed and optimized to address the challenge of exploiting plant byproducts through the recovery of targeted bioactive compounds, such as phenolic acids and flavonoids.

In the current study, UAE and MAE were optimized to acquire extracts with high phenolic content by implementing two-level full factorial and Box–Behnken designs. Methanol:water 4:1 % *v/v* was the extraction system in both techniques. In UAE, the higher TPC was achieved at 15 min, 8 s ON-5 s OFF sonication pulse, 35 mL g^−1^, 600 W and 35 °C, while MAE showed higher extraction efficiency at 20 min, 58 °C, 16 mL g^−1^, and 50 W. Comparing the two optimized methods in terms of TPC, MAE provided the higher yields and also the extracts with the higher antioxidant activity, whilst both optimal extracts presented high antiradical activity. The developed and validated LC-MS/MS method showed that the peach byproducts extracts contained significant amounts of chlorogenic acid. Based on LC-MS/MS results, the concentration of chlorogenic acid was higher when UAE was implemented. The higher values of chlorogenic acid may be attributed to the higher power of UAE, which may promote the release of chlorogenic acid if bound in more complex forms, and, at the same time, lower UAE temperature. On the other hand, the higher temperature of MAE appeared to not affect naringenin, since flavonoids are less thermolabile than phenolic acids.

To sum up, UAE and MAE could frame an attractive alternative approach to conventional extraction techniques for the generation of bioactive high-quality extracts.

## Figures and Tables

**Table 1:** Optimal values of the extraction factors.

Extraction Factors	Optimal Values	
	UAE	MAE
Extraction solvent (% *v/v*)	Methanol:Water 4:1	Methanol:Water 4:1
Extraction time (min)	15	20
Pulse sequence ON mode (s)	8	-
Pulse sequence OFF mode (s)	5	-
Extraction temperature (°C)	35	58
Solvent/material ratio (mL g^−1^)	35	16
US or MW power (W)	600	50
TPC at optimal conditions (mg of GAE g^−1^ dry sample) (±stdev), n = 3 ^1^	2.72 ± 0.30 ^b^	3.067 ± 0.027 ^a^

^1:^ Number of replicates; ^a,b^: Values with different letters differ significantly (*p*-value ≤ 0.05).

**Table 2:** Antiradical activity and reducing/antioxidant power of optimal UAE and MAE peach byproduct extracts.

Extract Type	mg TE·g^−1^ Dry Sample (t = 5 min)	mg TE·g^−1^ Dry Sample (Τ_plateau_ = 3 h)	mg Fe(II)·g^−1^ Dry Sample
UAE_optimal_	5.70 (±0.41) ^1,a^	9.84 (±0.95) ^a^	2.534 (±0.066) ^b^
MAE_optimal_	6.06 (±0.48) ^a^	9.83 (±0.95) ^a^	2.742 (±0.075) ^a^

^1^ Values are presented as mean (±standard deviation), (N = 3); ^a,b^ Means per column denoted by a common superscript letter are not significantly different according to Tukey’s test at 5% level of significance.

**Table 3:** Chlorogenic acid and naringenin concentrations in UAE and MAE dry peach byproduct extracts for 2^3^ design, BBD design, and optimal conditions.

Extraction Method	Run	Chlorogenic Acid (μg·g^−1^ Dry Sample)	Naringenin (μg·g^−1^ Dry Sample)
2^3^ design			
UAE	5	289.1 ± 4.0 ^1,c^	7.33 ± 0.28 ^c^
	6	284 ± 10 ^c^	6.89 ± 0.17 ^c^
MAE	3	285 ± 21 ^c^	6.27 ± 0.21 ^d^
	7	348.5 ± 7.7 ^a^	7.98 ± 0.35 ^b^
BBD design			
UAE	3	231.2 ± 5.9 ^d^	5.969 ± 0.039 ^e,f^
	11	314.4 ± 4.2 ^b^	7.71 ± 0.36 ^b,c^
MAE	7	290.4 ± 8.5 ^c^	6.02 ± 0.27 ^d,e^
	9	235.3 ± 7.8 ^d^	5.92 ± 0.29 ^d,f^
Optimal conditions			
UAE	optimal	351.1 ± 8.9 ^a^	7.13 ± 0.36 ^c^
ΜAE	optimal	295 ± 12 ^c^	9.42 ± 0.32 ^a^

^1^ Values are presented as mean (±standard deviation), (N = 3); ^a–f^ Means per column and denoted by a common superscript letter are not significantly different according to Tukey’s test at 5% level of significance.

**Table 4:** Real and coded values of extraction factors.

2^3^ Full Factorial Design			
Coded values	−1	0	+1
Extraction time (X_1_, min)	5	-	25
Pulse sequence ON (UAE) (X_2_, s)	10	-	30
Extraction temperature (MAE) (X_2_, °C)	50	-	70
Solvent/material ratio (X_3_, mL g^−1^)	20	-	50
Box–Behnken design			
Coded values for UAE	−1	0	+1
Extraction time (X_1_, min)	15	20	25
Pulse sequence ON (X_2_, s)	10	15	20
Solvent/material ratio (X_3_, mL g^−1^)	15	25	35
Coded values for MAE	−1	0	+1
Extraction time (X_1_, min)	15	20	25
Extraction temperature (X_2_, °C)	60	65	70
Solvent/material ratio (X_3_, mL g^−1^)	15	20	25

**Table 5:** Molecular structures, formulas, and characteristic features for the LC-MS/MS analysis of the targeted phenolic compounds and internal standard.

Compound	Molecular Structure ^2^	MolecularFormula	Rt ^1^ (min)	MRM Transition (*m/z*)	Product Ion	DP ^1^ (V)	EP ^1^ (V)	CEP ^1^ (V)	CE ^1^ (eV)	CXP ^1^ (V)
Chlorogenic acid		C_16_H_18_O_9_	1.9	353.0 > 191.1	qualifier	−50	−4.5	−14	−26	−4
				353.0 > 84.8	quantifier	−50	−4.5	−14	−56	0
Naringenin	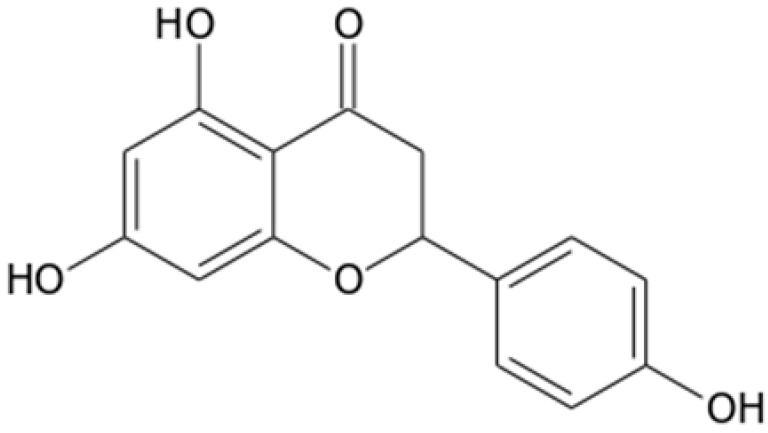	C_15_H_12_O_5_	6.5	270.9 > 151.0	qualifier	−55	−6.5	−14	−26	−2
				270.9 > 119.1	quantifier	−55	−6.5	−14	−34	−2
4-Chloro-4′-hydroxybenzophenone (IS)	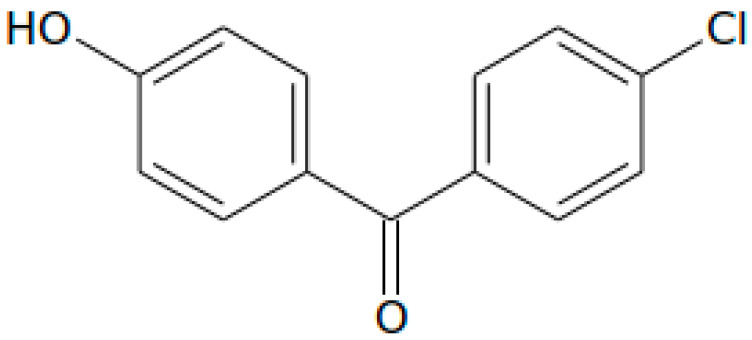	C_13_H_9_ClO_2_	7.2	231.0 > 195.0	qualifier	−70	−9	−12	−32	0
				231.0 > 92.0	quantifier	−70	−9	−12	−32	−2

^1^ Rt: retention time; *m/z*: mass-to-charge ratio; DP: declustering potential; EP: entrance potential; CEP: collision cell entrance potential; CE: collision energy; CXP: collision cell exit potential. ^2^ Graphics of molecular structures were formulated using the chemical structures editor GChemPaint, v. 0.14.17 (under the terms of the GNU General Public License as published by the Free Software Foundation, Inc., Boston, MA, USA).

## Data Availability

Not applicable.

## Supplementary Materials

The following supporting information can be downloaded at: https://www.mdpi.com/article/10.3390/molecules28020518/s1, Table S1: UAE and MAE randomized experimental runs and TPC of dry peach byproducts for 2^3^ and BBD designs; Table S2: ANOVA table of 2^3^ designs for UAE and MAE of peach byproducts; Table S3: ANOVA table of BBD designs for UAE and MAE of peach byproducts; Table S4: Predicted and observed TPC of peach byproducts at the experimental combinations proposed as optimal by the BBD models; Figure S1a,b: Contour plots of: (a) UAE extraction time vs. pulse sequence ON; (b) UAE extraction time vs. solvent/material ratio; (c) UAE pulse sequence ON vs. solvent/material ratio; (d) MAE extraction time vs. extraction temperature; (e) MAE extraction time vs. solvent/material ratio; (f) MAE extraction temperature vs. solvent/material ratio; Table S5: Analytical figures of merit of LC-MS/MS method for chlorogenic acid and naringenin quantitation in peach byproduct extracts; Table S6: Matrix effect for chlorogenic acid and naringenin in peach byproduct extracts as determined by LC-MS/MS; Table S7: Process recovery of chlorogenic acid and naringenin in UAE and MAE peach byproduct extracts as determined by LC-MS/MS; Table S8: Precision expressed as repeatability and reproducibility, and accuracy of LC-MS/MS method for chlorogenic acid and naringenin determination in peach byproduct extracts; Table S9: Summary of stability results for analytes and internal standard using the proposed LC- MS/MS method.

## References

[1] Marcillo-Parra V., Tupuna-Yerovi D.S., González Z., Ruales J. (2021). Encapsulation of bioactive compounds from fruit and vegetable by-products for food application—A review. Trends Food Sci. Technol..

[2] Torres-León C., Ramírez-Guzman N., Londoño-Hernandez L., Martinez-Medina G.A., Díaz-Herrera R., Navarro-Macias V., Alvarez-Pérez O.B., Picazo B., Villarreal-Vázquez M., Ascacio-Valdes J. (2018). Food Waste and Byproducts: An Opportunity to Minimize Malnutrition and Hunger in Developing Countries. Front. Sustain. Food Syst..

[3] Freitas L.C., Barbosa J.R., da Costa A.L.C., Bezerra F.W.F., Pinto R.H.H., Junior R.N.C. (2021). From waste to sustainable industry: How can agro-industrial wastes help in the development of new products?. Resour. Conserv. Recycl..

[4] Swallah M.S., Sun H., Affoh R., Fu H., Yu H. (2020). Antioxidant Potential Overviews of Secondary Metabolites (Polyphenols) in Fruits. Int. J. Food Sci..

[5] Jideani A.I.O., Silungwe H., Takalani T., Omolola A.O., Udeh H.O., Anyasi T.A. (2021). Antioxidant-rich natural fruit and vegetable products and human health. Int. J. Food Prop..

[6] Nanaki E.A., Koroneos C.J. (2018). Sustainable Peach Compote Production: A Life Cycle Thinking Approach. Sustainability.

[7] Louka P., Kalatzis N., Marianos N. (2022). SmartPeach: Smart Farming Practices Enhance the Adaptation of Peach Crops to Climate Change. Chem. Proc..

[8] Ran X., Zhang M., Wang Y., Adhikari B. (2019). Novel technologies applied for recovery and value addition of high value compounds from plant byproducts: A review. Crit. Rev. Food Sci. Nutr..

[9] Lourenço S.C., Moldão-Martins M., Alves V.D. (2019). Antioxidants of Natural Plant Origins: From Sources to Food Industry Applications. Molecules.

[10] Rodríguez García S.L., Raghavan V. (2022). Green extraction techniques from fruit and vegetable waste to obtain bioactive compounds—A review. Crit. Rev. Food Sci. Nutr..

[11] Yusoff I.M., Taher Z.M., Rahmat Z., Chua L.S. (2022). A review of ultrasound-assisted extraction for plant bioactive compounds: Phenolics, flavonoids, thymols, saponins and proteins. Food Res. Int..

[12] Bagade S.B., Patil M. (2021). Recent Advances in Microwave Assisted Extraction of Bioactive Compounds from Complex Herbal Samples: A Review. Crit. Rev. Anal. Chem..

[13] More P.R., Arya S.S. (2021). Intensification of bio-actives extraction from pomegranate peel using pulsed ultrasound: Effect of factors, correlation, optimization and antioxidant bioactivities. Ultrason. Sonochemistry.

[14] Bezerra M.A., Santelli R.E., Oliveira E.P., Villar L.S., Escaleira L.A. (2008). Response surface methodology (RSM) as a tool for optimization in analytical chemistry. Talanta.

[15] Vázquez-Espinosa M., González de Peredo A.V., Ferreiro-González M., Barroso C.G., Palma M., Barbero G.F., Espada-Bellido E. (2019). Optimizing and comparing ultrasound-and microwave-assisted extraction methods applied to the extraction of antioxidant capsinoids in peppers. Agronomy.

[16] Agarwal C., Máthé K., Hofmann T., Csóka L. (2018). Ultrasound-assisted extraction of cannabinoids from Cannabis sativa L. optimized by response surface methodology. J. Food Sci..

[17] Santos-Zea L., Gutierrez-Uribe J.A., Benedito J. (2021). Effect of Solvent Composition on Ultrasound-Generated Intensity and Its Influence on the Ultrasonically Assisted Extraction of Bioactives from Agave Bagasse (Agave salmiana). Food Eng. Rev..

[18] Tsiaka T., Fotakis C., Lantzouraki D.Z., Tsiantas K., Siapi E., Sinanoglou V.J., Zoumpoulakis P. (2020). Expanding the Role of Sub-Exploited DOE-High Energy Extraction and Metabolomic Profiling towards Agro-Byproduct Valorization: The Case of Carotenoid-Rich Apricot Pulp. Molecules.

[19] Xie P., Huang L., Zhang C., You F., Zhang Y. (2015). Reduced pressure extraction of oleuropein from olive leaves (*Olea europaea L*.) with ultrasound assistance. Food Bioprod. Process..

[20] Zhou T., Xu D.-P., Lin S.-J., Li Y., Zheng J., Zhou Y., Zhang J.-J., Li H.-B. (2017). Ultrasound-Assisted Extraction and Identification of Natural Antioxidants from the Fruit of Melastoma sanguineum Sims. Molecules.

[21] Foujdar R., Bera M.B., Chopra H.K. (2020). Optimization of process variables of probe ultrasonic-assisted extraction of phenolic compounds from the peel of Punica granatum Var. Bhagwa and it’s chemical and bioactivity characterization. J. Food Process. Preserv..

[22] Rodsamran P., Sothornvit R. (2019). Extraction of phenolic compounds from lime peel waste using ultrasonic-assisted and microwave-assisted extractions. Food Biosci..

[23] Azaroual L., Liazid A., Mansouri F.E., Brigui J., Ruíz-Rodriguez A., Barbero G.F., Palma M. (2021). Optimization of the Microwave-Assisted Extraction of Simple Phenolic Compounds from Grape Skins and Seeds. Agronomy.

[24] Wang J., Ma H., Pan Z., Qu W. (2017). Sonochemical effect of flat sweep frequency and pulsed ultrasound (FSFP) treatment on stability of phenolic acids in a model system. Ultrason. Sonochemistry.

[25] Alara O.R., Abdurahman N.H. (2019). Microwave-assisted extraction of phenolics from Hibiscus sabdariffa calyces: Kinetic modelling and process intensification. Ind. Crops Prod..

[26] Zhao C.N., Zhang J.J., Li Y., Meng X., Li H.B. (2018). Microwave-assisted extraction of phenolic compounds from Melastoma sanguineum fruit: Optimization and identification. Molecules.

[27] Dzah C.S., Duan Y., Zhang H., Wen C., Zhang J., Chen G., Ma H. (2020). The effects of ultrasound assisted extraction on yield, antioxidant, anticancer and antimicrobial activity of polyphenol extracts: A review. Food Biosci..

[28] Flórez N., Conde E., Domínguez H. (2015). Microwave assisted water extraction of plant compounds. J. Chem. Technol. Biotechnol..

[29] Coelho E., Rocha M.A.M., Saraiva J.A., Coimbra M.A. (2014). Microwave superheated water and dilute alkali extraction of brewers’ spent grain arabinoxylans and arabinoxylo-oligosaccharides. Carbohydr. Polym..

[30] Xiaokang W., Lyng J.G., Brunton N.P., Cody L., Jacquier J.-C., Harrison S.M., Papoutsis K. (2020). Monitoring the effect of different microwave extraction parameters on the recovery of polyphenols from shiitake mushrooms: Comparison with hot-water and organic-solvent extractions. Biotechnol. Rep..

[31] Bento C., Gonçalves A.C., Silva B., Silva L.R. (2022). Peach (Prunus Persica): Phytochemicals and Health Benefits. Food Rev. Int..

[32] Christofi M., Pavlou A., Lantzouraki D.Z., Tsiaka T., Myrtsi E., Zoumpoulakis P., Haroutounian S.A., Mauromoustakos A., Biliaderis C.G., Manganaris G.A. (2022). Profiling carotenoid and phenolic compounds in fresh and canned fruit of peach cultivars: Impact of genotype and canning on their concentration. J. Food Compos. Anal..

[33] Mihaylova D., Popova A., Desseva I., Petkova N., Stoyanova M., Vrancheva R., Slavov A., Slavchev A., Lante A. (2021). Comparative Study of Early- and Mid-Ripening Peach (Prunus persica L.) Varieties: Biological Activity, Macro-, and Micro- Nutrient Profile. Foods.

[34] Bkhairia I., Salem R.B.S.B., Nasri R., Jridi M., Ghorbel S., Nasri M. (2016). In-vitro antioxidant and functional properties of protein hydrolysates from golden grey mullet prepared by commercial, microbial and visceral proteases. J. Food Sci. Technol..

[35] Mirzaei M., Aminlari M., Hosseini E. (2016). Antioxidant, ACE-Inhibitory and Antimicrobial Activities of Kluyveromyces marxianus Protein Hydrolysates and Their Peptide Fractions. Funct. Foods Health Dis..

[36] Zhang L., Zhu C., Liu X., Su E., Cao F., Zhao L. (2022). Study on Synergistic Antioxidant Effect of Typical Functional Components of Hydroethanolic Leaf Extract from Ginkgo Biloba In Vitro. Molecules.

[37] Joshi T., Deepa P.R., Sharma P.K. (2022). Effect of Different Proportions of Phenolics on Antioxidant Potential: Pointers for Bioactive Synergy/Antagonism in Foods and Nutraceuticals. Proc. Natl. Acad. Sci. India Sect. B Biol. Sci..

[38] Lantzouraki D.Z., Sinanoglou V.J., Zoumpoulakis P., Proestos C. (2016). Comparison of the Antioxidant and Antiradical Activity of Pomegranate (*Punica granatum L*.) by Ultrasound-Assisted and Classical Extraction. Anal. Lett..

[39] Kedare S.B., Singh R.P. (2011). Genesis and development of DPPH method of antioxidant assay. J. Food Sci. Technol..

[40] Wołosiak R., Drużyńska B., Derewiaka D., Piecyk M., Majewska E., Ciecierska M., Worobiej E., Pakosz P. (2022). Verification of the Conditions for Determination of Antioxidant Activity by ABTS and DPPH Assays—A Practical Approach. Molecules.

[41] Rossato S.B., Haas C., Raseira M.D.C.B., Moreira J.C.F., Zuanazzi J.Â.S. (2009). Antioxidant Potential of Peels and Fleshes of Peaches from Different Cultivars. J. Med. Food.

[42] Tomás-Barberán F.A., Gil M.I., Cremin P., Waterhouse A.L., Hess-Pierce B., Kader A.A. (2001). HPLC− DAD− ESIMS analysis of phenolic compounds in nectarines, peaches, and plums. J. Agric. Food Chem..

[43] Dong Z., Wang R., Wang M., Meng Z., Wang X., Han M., Guo Y., Wang X. (2022). Preparation of Naringenin Nanosuspension and Its Antitussive and Expectorant Effects. Molecules.

[44] Liu J., Zhang X., Tian J., Li Y., Liu Q., Chen X., Feng F., Yu X., Yang C. (2022). Multiomics analysis reveals that peach gum colouring reflects plant defense responses against pathogenic fungi. Food Chem..

[45] Li M., Fokkink R., Ni Y., Kleijn J.M. (2019). Bovine beta-casein micelles as delivery systems for hydrophobic flavonoids. Food Hydrocoll..

[46] Nunes N.M., Coelho Y.L., Castro J.S., Vidigal M.C.T.R., Mendes T.A.O., da Silva L.H.M., Pires A.C.S. (2020). Naringenin-lactoferrin binding: Impact on naringenin bitterness and thermodynamic characterization of the complex. Food Chem..

[47] Vargas F., Canudas N., Miranda M.A., Boscar F. (1993). PHOTODEGRADATION AND in vitro PHOTOTOXICITY OF FENOFIBRATE, A PHOTOSENSITIZING ANTI-HYPEIUIPOPROTEINEMIC DRUG. Photochem. Photobiol..

[48] FDA US Food and Drug Administration, Bioanalytical Method Validation—Guidance for Industry. https://www.fda.gov/files/drugs/published/Bioanalytical-Method-Validation-Guidance-for-Industry.pdf.

[49] Tan A., Awaiye K. (2013). Use of Internal Standards in LC-MS Bioanalysis. Handbook of LC-MS Bioanalysis.

[50] Saidani F., Giménez R., Aubert C., Chalot G., Betrán J.A., Gogorcena Y. (2017). Phenolic, sugar and acid profiles and the antioxidant composition in the peel and pulp of peach fruits. J. Food Compos. Anal..

[51] Koprivica M.R., Trifković J.Đ., Dramićanin A.M., Gašić U.M., Akšić M.M.F., Milojković-Opsenica D.M. (2018). Determination of the phenolic profile of peach (*Prunus persica* L.) kernels using UHPLC–LTQ OrbiTrap MS/MS technique. Eur. Food Res. Technol..

[52] Tagkouli D., Tsiaka T., Kritsi E., Soković M., Sinanoglou V.J., Lantzouraki D.Z., Zoumpoulakis P. (2022). Towards the Optimization of Microwave-Assisted Extraction and the Assessment of Chemical Profile, Antioxidant and Antimicrobial Activity of Wine Lees Extracts. Molecules.

[53] Lantzouraki D.Z., Sinanoglou V.J., Zoumpoulakis P.G., Glamočlija J., Ćirić A., Soković M., Heropoulos G., Proestos C. (2014). Antiradical–antimicrobial activity and phenolic profile of pomegranate (*Punica granatum* L.) juices from different cultivars: A comparative study. RSC Adv..

[54] Lantzouraki D.Z., Tsiaka T., Soteriou N., Asimomiti G., Spanidi E., Natskoulis P., Gardikis K., Sinanoglou V.J., Zoumpoulakis P. (2020). Antioxidant Profiles of Vitis vinifera L. and Salvia triloba L. Leaves Using High-Energy Extraction Methodologies. J. AOAC Int..

[55] Tsiaka T., Kritsi E., Lantzouraki D.Z., Christodoulou P., Tsigrimani D., Strati I.F., Sinanoglou V.J., Zoumpoulakis P. (2022). Assessing the Phytochemical Profile and Potential of Traditional Herbal Infusions against Aldose Reductase through In Silico Studies and LC-MS/MS Analysis. Appl. Sci..

[56] ICH International Council for Harmonisation of Technical Requirements for Pharmaceuticals for Human Use, ICH Harmonised Guidelines M10—Bioanalytical Method Validation (Draft Version). https://www.ema.europa.eu/en/documents/scientific-guideline/draft-ich-guideline-m10-bioanalytical-method-validation-step-2b_en.pdf.

[57] EMA European Medicines Agency, Guideline of Bioanalytical Method Validation. https://www.ema.europa.eu/en/documents/scientific-guideline/guideline-bioanalytical-method-validation_en.pdf.

[58] Faccin H., Viana C., Nascimento P.C.D., Bohrer D., de Carvalho L.M. (2016). Study of ion suppression for phenolic compounds in medicinal plant extracts using liquid chromatography–electrospray tandem mass spectrometry. J. Chromatogr. A.

[59] Cittan M., Çelik A. (2018). Development and Validation of an Analytical Methodology Based on Liquid Chromatography–Electrospray Tandem Mass Spectrometry for the Simultaneous Determination of Phenolic Compounds in Olive Leaf Extract. J. Chromatogr. Sci..

[60] Liu Z., Tu M.-J., Zhang C., Jilek J.L., Zhang Q.-Y., Yu A.-M. (2019). A Reliable LC-MS/MS Method for the Quantification of Natural Amino Acids in Mouse Plasma: Method Validation and Application to a Study on Amino Acid Dynamics during Hepatocellular Carcinoma Progression. J. Chromatogr. B.

