# Supplementary material for: Optimization of Ultrasound- and Microwave-Assisted Extraction for the Determination of Phenolic Compounds in Peach Byproducts Using Experimental Design and Liquid Chromatography–Tandem Mass Spectrometry
Source: Molecules. 2023 Jan 5;28(2):518. doi: 10.3390/molecules28020518 (PMC9867053; doi:10.3390/molecules28020518)
Supplement: Supplementary file 1 [file molecules-28-00518-s001.zip › molecules-2082245-supplementary.pdf]

molecules

MDPI

Supplementary Materials

Table S1. UAE and MAE randomized experimental runs and TPC of dry peach byproducts for 2^3 and BBD designs.

Standard Run	Coded Combinations (x_1, x_2, x_3)	TPC of UAE (mg GAE/g dry sample) (±stdev), n=3[1]	TPC of MAE (mg GAE/g dry sample) (±stdev), n=3
		2^3 design	
5	+1,-1,-1	2.08±0.23	2.09±0.14
3	-1,+1,-1	1.82±0.16	1.87±0.10
6	+1,-1,+1	1.69±0.23	1.93±0.19
8	+1,+1,+1	1.835±0.088	2.271±0.096
4	-1,+1,+1	1.368±0.010	1.82±0.17
1	-1,-1,-1	1.92±0.23	1.903±0.086
7	+1,+1,-1	1.88±0.14	2.61±0.16
2	-1,-1,+1	1.824±0.093	2.23±0.18
		BBD design	
11	0,-1,+1	1.966±0.075	2.001±0.063
9	0,-1,-1	1.699±0.066	2.894±0.035
4	+1,+1,0	1.610±0.048	2.331±0.055
3	-1,+1,0	1.575±0.044	2.032±0.017
8	+1,0,+1	1.609±0.055	2.094±0.070
10	0,+1,-1	1.704±0.036	2.354±0.092
5	-1,0,-1	1.606±0.045	2.018±0.081
6	+1,0,-1	1.608±0.073	2.097±0.078
7	-1,0,+1	1.762±0.064	1.954±0.071
13	0,0,0	1.694±0.048	2.139±0.046
2	+1,0,0	1.723±0.067	2.053±0.060
12	0,+1,+1	1.691±0.051	2.039±0.057
1	-1,-1,0	1.599±0.055	2.267±0.034
16	0,0,0	1.586±0.056	2.183±0.076
15	0,0,0	1.608±0.046	2.272±0.094
14	0,0,0	1.691±0.069	2.245±0.067

[1] The number of replicates

Table S2. ANOVA table of 2^3 designs for UAE and MAE of peach byproducts.

	UAE		
**Model terms**	**Sum of Squares (SS)**	***F*-value**	***p*-value**
x_1	0.037	1.74	0.28
x_2	0.047	2.23	0.23
x_3	0.12	5.70	0.09
x_1*x_2	0.031	1.46	0.31
**Total SS (Degrees of Freedom)**			0.30
**R^2**			0.79
**R^2_{adj}**			0.50

	MAE		
**Model terms**	**Sum of Squares (SS)**	***F*-value**	***p*-value**
x_2	0.15	3.83	0.15
x_3	0.10	2.60	0.21
x_1*x_2	0.49	12.41	0.04[a]
x_1*x_3	0.27	6.76	0.09
**Total SS (Degrees of Freedom)**			1.14
**R^2**			0.90
**R^2_{adj}**			0.76

[a] Terms with p-value≤0.05

Table S3. ANOVA table of BBD designs for UAE and MAE of peach byproducts.

UAE			
**Model terms**	**Sum of Squares (SS)**	***F*-value**	***p*-value**
x_1(Q)[1]	0.019	8.12	0.02[a]
x_2(L)[2]	0.021	8.94	0.02[a]
x_2(Q)	0.010	4.41	0.07
x_3(L)	0.021	9.16	0.02[a]
x_3(Q)	0.019	8.46	0.02[a]
x_1(L)x_2(Q)	0.011	4.60	0.07
x_1(L)x_3(L)	0.006	2.62	0.15
x_2(L)x_3(L)	0.020	8.50	0.02[a]
**Total SS (Degrees of Freedom)**			0.14
**$MS_{residual}$**			0.0023
**R^2**			0.887
**R^2_{adj}**			0.757

MAE			
**Model terms**	**Sum of Squares (SS)**	***F*-value**	***p*-value**
x_1(L)	0.008	2.11	0.24
x_1(Q)	0.102	28.45	0.01[a]
x_2(L)	0.009	2.39	0.22
x_2(Q)	0.059	16.29	0.03[a]
x_3(L)	0.090	24.99	0.01[a]
x_1(L)x_2(L)	0.066	18.21	0.02[a]
x_1(L)x_2(Q)	0.002	0.62	0.49
x_1(Q)x_2(L)	0.037	10.28	0.05[a]
x_1(L)x_3(L)	0.001	0.26	0.64
x_1(Q)x_3(L)	0.163	45.32	0.01[a]
x_2(L)x_3(L)	0.0003	23.20	0.02[a]
**Total SS (Degrees of Freedom)**			0.77
**$MS_{residual}$**			0.0028
**R^2**			0.986
**R^2_{adj}**			0.946

[1] L: linear terms, [2] Q: quadratic terms, [a] Terms with p-value≤0.05

Table S4. Predicted and observed TPC of peach byproducts at the experimental combinations proposed as optimal by the BBD models.

UAE	Extraction time (min)	Pulse sequence ON mode (s)	Solvent/material ratio (mL/g)	Predicted TPC value (mg GAE/g dry sample)	Experimental TPC value (mg GAE/g dry sample) (±stdev) (±stdev), n=3[1]
Run A	20	8	35	2.00	2.28±0.14[a]
Run B	15	10	35	1.92	2.32±0.20[a]
Run C	15	8	35	2.02	2.72±0.30[a]
**MAE**	**Extraction time (min)**	**Extraction temperature (°C)**	**Solvent/material ratio (mL/g)**	**Predicted TPC value (mg GAE/g dry sample)**	**Experimental TPC value (mg GAE/g dry sample) (±stdev) (±stdev), n=3[1]**
Run A	20	58	16	3.021	3.067±0.027[b]
Run B	20	60	16	3.22	2.98±0.22[b]
Run C	16	58	16	2.95	2.83±0.12[b]

[a],[b]: Values with same letter, in each extraction technique, do not differ significantly (p-value≤0.05); [1]: the number of replicates

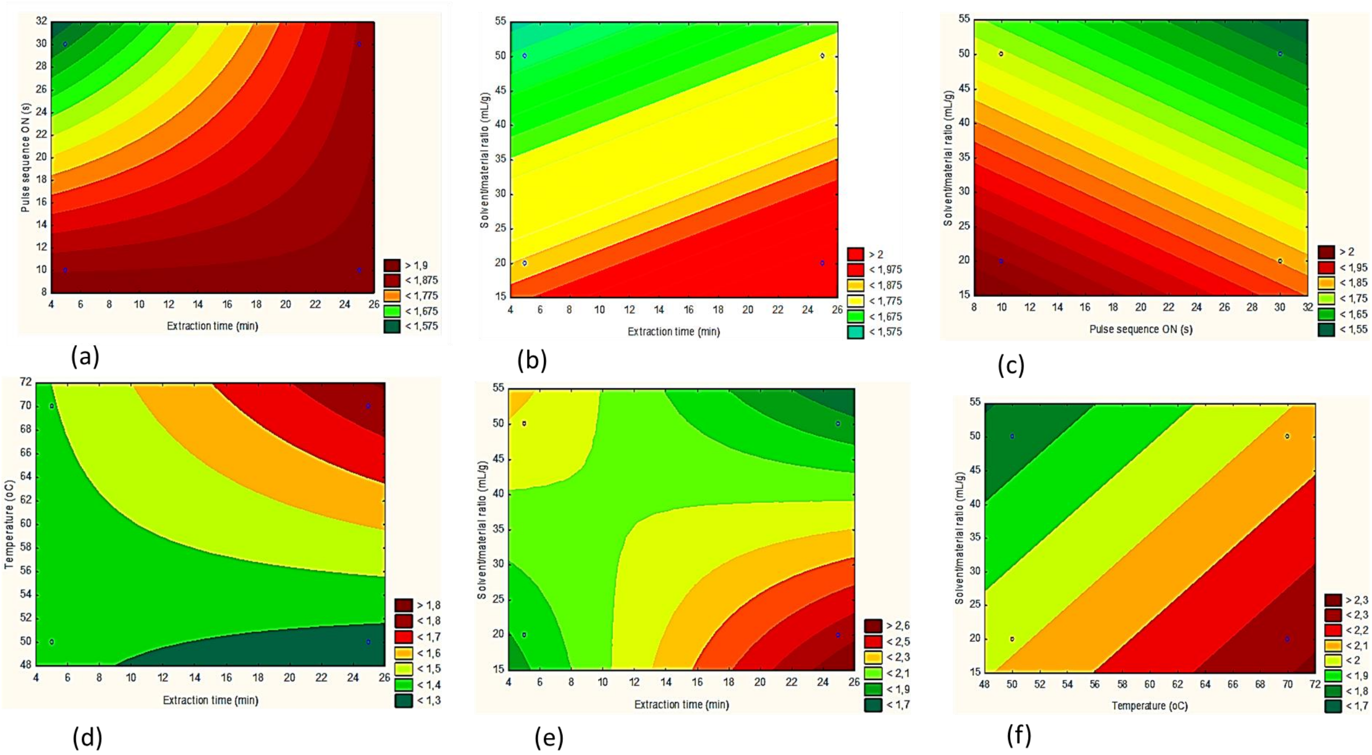

Figure S1. Contour plots of: (a) UAE extraction time vs pulse sequence ON; (b) UAE extraction time vs solvent/material ratio; (c) UAE pulse sequence ON vs solvent/material ratio; (d) MAE extraction time vs extraction temperature; (e) MAE extraction time vs solvent/material ratio; (f) MAE extraction temperature vs solvent/material ratio

S1. HPLC-ESI-QqQ-MS/MS(MRM) Method Validation for the determination of targeted phenolic compounds

The validation of the method was performed as per European Medicines Agency (EMA) [1], US Food and Drug Administration (FDA) [2] and International Council for Harmonisation (ICH) [3] and in accordance with other published works that include plant matrices [4]

S1.1. Selectivity

Selectivity was assessed by comparing MRM chromatograms for each standard compound attained from six runs of matrix samples with those post-spiked with low concentration equivalent to the limits of quantitation (LOQs) for each analyte, and at nominal concentration used for internal standard (1.0 $\mu g \cdot mL^{-1}$). No interference peak was detected at the retention times of either chlorogenic acid or naringenin or internal standard indicating acceptable degree of method selectivity.

S1.2. Calibration curves and linearity

Pre- and post-spiked matrix-matched calibration curves for chlorogenic acid and naringenin were constructed by using pooled mixes of the optimal UAE and MAE dry extracts in ratio of 1:1 (w/w). Pre-spiked standard curve was applied for the quantitation process, while both matrix-matched curves were used for assessing process recovery. Spiked calibration curves were constructed by analyzing 8 different calibrators for each analyte, specifically from 0.35 to 2.38, and from 0.0071 to 0.048 $\mu g \cdot mL^{-1}$ for chlorogenic acid and naringenin, respectively. The final concentration of dry extract material for each extract type was set at 15 mg dry matter·mL^{-1} in 1:1 (v/v) mixture of methanol and 0.1% (v/v) aqueous formic acid. Additionally, external calibration curves of standards in solvents were acquired at concentration range corresponding to matrix-matched curves in order to determine matrix effect along with the use of post-spike curve, for each analyte. Internal standard concentration was 1.0 $\mu g \cdot mL^{-1}$ in all calibration curves. The linearity of all curves was acceptable with coefficient of determination (R^2)≥0.993, and none of the calibration points had to be excluded for establishing the linear regressions as the absolute value of deviation from the nominal value was lower than 15%. The analytical figures of merit for the pre-spiked calibration curves employed for quantitation are plainly presented in Table S5.

S1.3. Limits of detection and quantitation

Limit of detection (LOD) and limit of quantitation (LOQ) were calculated by preparing two additional calibration curves around the concentration area of the lowest spiking levels for each analyte (Table S5). LOD and LOQ values were delivered by determining $(3.3 \times s_b/a) \times C_{IS}$ concentration ($\mu g \cdot mL^{-1}$) and $(10 \times s_b/a) \times C_{IS}$ concentration ($\mu g \cdot mL^{-1}$), respectively. In these equations, a corresponds to curve slope, s_b to intercept standard deviation and C_{IS} is equal to the concentration of IS (1.0 $\mu g \cdot mL^{-1}$). On the whole, naringenin presented significantly lower LOD and LOQ values than chlorogenic acid (Table S5) due to better signal response of the analyte, lower background noise and less matrix interferences in the mass spectra.

Table S5. Analytical figures of merit of LC-MS/MS method for chlorogenic acid and naringenin quantitation in peach byproduct extracts.

Analytical figures of merit	Chlorogenic acid	Naringenin
Linear range for calibration curves (μg·mL^{-1})	0.36–2.4	0.0071–0.048
Slope, a (±s_a) (N = 3)[1]	2.377(±0.076)[2]	10.97(±0.37)
Intercept, b (±s_b) (N = 3)	-0.02(±0.11)	0.041(±0.011)
Coefficient of determination, R^2	0.994	0.993
LOD (μg·mL^{-1}) (N = 3)	0.060	0.0026
LOQ (μg·mL^{-1}) (N = 3)	0.18	0.0080
Concentration range of calibration curves for LOD/LOQ (μg·mL^{-1})	0.023–0.60	0.00048–0.012

[1]N: Number of quality control samples replicates; [2] Values are presented as mean(±standard deviation)

S1.4. Matrix effect

Matrix effect (ME) accounts for MS signal suppression or enhancement that may occur during the ionization of the molecules caused by co-eluting or other process-added constituents. In our study, ME was assessed by taking into account the naturally pre-existing concentration of each examined analyte in the neat matrix sample solution due to the lack of blank matrix, following Equation S1 [5]. The ME was determined in a low, medium and high concentration level (Low QC-LQC, Medium QC-MQC, High QC-HQC) for the post-spiked phenolic compounds (Table S6).

$$ME(\%) = (Area_{POST\text{-}SPIKED} - Area_{MATRIX} - Area_{ANALYTE}) \times 100 / Area_{ANALYTE} \quad (S1)$$

In Equation S1, $Area_{POST\text{-}SPIKED}$ stands for the peak area of the analyte in a matrix-matched quality control sample where the examined analyte was added just after the extraction process and the sequential step for further treatment before LC-MS analysis, $Area_{MATRIX}$ for the peak area of the analyte in a neat matrix solution, and $Area_{ANALYTE}$ is the analyte peak area in pure solvents. As seen in Table S6, a significant signal suppression (ME%<0) was observed at the three concentration levels for both phenolic compounds. Such suppression degree is likely caused by the concentration of dissociated formate anions, which lowers the ionization of phenolic compounds in negative electrospray ionization mode. Formic acid possesses a low pKa value that can also promote the ionization of interfering chemical species in the analysis of various plants [6]. Since the matrix effect could interfere with accuracy in quantitation results, matrix-matched calibration curves were constructed for each analyte to compensate the suppression phenomena and avoid erroneous results. Overall, matrix suppression was consistent between quality control (QC) levels, especially for chlorogenic acid, while standard deviations were <15% as observed in our data (Table S6).

Table S6. Matrix effect for chlorogenic acid and naringenin in peach byproduct extracts as determined by LC-MS/MS.

Analyte	Spike level (μg·mL^{-1})	Matrix effect, ME (%) (N=3)[1]
Chlorogenic acid	0.60	−61.3(±1.3)[2]
	1.2	−61.7(±3,2)
	2.0	−61.6(±2.6)
Naringenin	0.012	−40.3(±2.3)
	0.024	−49.0(±4.0)
	0.040	−47.3(±2.8)

[1] N: Number of quality control samples replicates; [2] Values are presented as mean(±standard deviation)

S1.5. Process recovery, precision and accuracy

Process recovery was determined by comparing the computed concentration of analyte in quality control samples spiked before and after the extraction procedure followed by further treatment prior LC-MS injection. Therefore, process recovery encompasses the losses of analytes occurring during extraction procedure and extract treatment for LC-MS analysis. In parallel, due to the presence of endogenous phenolic compounds in peach byproduct samples, recovery calculation was corrected with the corresponding peak area in the original matrix. Recovery was calculated by Equation S2 separately for the the two extraction methods, i.e. UAE and MAE. For the case of chlorogenic acid 150 μg standard were added per gram of lyophilized peach byproduct, while for naringenin the respective concentration was set at 5 μg·g^{-1} for the preparation of spiked samples.

$$\text{Process recovery}(\%) = (\text{Area}_{\text{PRE-SPIKED}} - \text{Area}_{\text{MATRIX}}) \times 100 / (\text{Area}_{\text{POST-SPIKED}} - \text{Area}_{\text{MATRIX}}) \quad \text{(S2)}$$

$\text{Area}_{\text{PRE-SPIKED}}$ represents the analyte peak area in a quality control sample where the analyte was added to the matrix just before the extraction step, $\text{Area}_{\text{MATRIX}}$ is the peak area of the analyte in naturally found in a neat matrix solution, and $\text{Area}_{\text{POST-SPIKED}}$ is the analyte peak area in a post-spiked matrix-matched quality control sample.

According to Table S7 process recoveries were similar (70.7–72.6%) for chlorogenic acid and naringenin concerning UAE extraction. For MAE extraction chlorogenic acid showed approximately 1.5-fold higher recovery compared with the UAE extract. On the contrary, MAE extraction of naringenin proven 1.5-fold less efficient than the UAE extraction. Overall, the two-step total sample preparation procedure, including peach byproduct extraction and the sequential treatment for LC-MS analysis, was adequate to achieve acceptable recovery for the studied compounds.

Table S7. Process recovery of chlorogenic acid and naringenin in UAE and MAE peach byproduct extracts as determined by LC-MS/MS.

Analyte	Extraction type	Process recovery (N=3)[1]
Chlorogenic acid	UAE	70.7(±1.6)[2]
	MAE	105.1(±2.0)
Naringenin	UAE	72.6(±1.7)
	MAE	48.7(±1.2)

[1] Values are presented as mean(±standard deviation); [2] N: Number of quality control samples replicates.

Three quality control samples at different concentration levels (LQC, MQC, HQC) and on three different days were measured in triplicate to assess the precision and accuracy of the method. Method precision was estimated in terms of intra- and inter-day assays. The relative standard deviation RSD (%) of QC samples data indicated the intra-day precision (repeatability) on a same day. For inter-day precision (reproducibility) analysis on three replicates of QC samples was carried out on three consecutive days. Accuracy was investigated by calculating the relative error (RE%), that is the percentage difference of the measured to the nominal value within an assay. The RSD (%) values for repeatability and reproducibility for chlorogenic acid and naringenin presented in Table S8 were found to be within acceptable limits. An accuracy in a margin of error of ±15% and a precision of ≤ 15% were accepted as valid. Altogether, the resulting fluctuations as depicted by RSD (%) did not exceed 6.2% for intra-assays, and 15% for inter-assays. Likewise, RE (%) values were <8.4% for both analytes (Table S8). These data indicated that the established method presented here was accurate and precise for the purposed quantitative analysis.

Table S8. Precision expressed as repeatability and reproducibility, and accuracy of LC-MS/MS method for chlorogenic acid and naringenin determination in peach byproduct extracts.

Analyte	QC concentration level (μg·mL^{-1})	Repeatability (RSD%) (N=3)[1]	Reproducibility (RSD%) (N=3; n=3[2])	Accuracy (RE%)(N=3)
Chlorogenic acid	0.60	5.30	13.55	2.07
	1.2	4.37	12.48	2.93
	2.0	5.65	12.66	3.22
Naringenin	0.012	6.18	8.92	8.36
	0.024	3.13	3.22	8.37
	0.040	4.13	5.69	1.60

[1] N: Number of QC replicates; [2] n: Number of consecutive days required for inter-day precision determination.

S1.6. Stability of standard compounds

Stability tests were performed for the targeted phenolic compounds and internal standard in matrix samples. In specifics, three QC levels (LQH, MQC, HQC) containing the target phenolic compounds were investigated in a short and a long time period assay, respectively. The stability of internal standard was evaluated at the concentration set for all standard and sample solutions used throughout the study (1 μg·mL^{-1}). Quality controls were analyzed in triplicate on the day of their preparation from stock solutions encompassing bench stability for 4 h at 20°C, and after 1 month upon storage at −20°C. Stability of compounds was reviewed on the basis (i) of accuracy for recovered concentrations by calculating the relative error (RE%), which is the percentage difference of the measured to the nominal value within an assay, and (ii) of the percentage change of measured concentrations between the two time points (% change). T-tests were also used to compare the means of concentrations and reveal whether % change was considerable concerning chemical stability. Probabilities lower than 0.05 (P<0.05) were

considered statistically significant. The general acceptance criteria we took into account for evaluation were: RSD (%): ≤20%; RE (%): ±10%; % Change ≤15%. Results are summarized in Table S9.

According to our study, naringenin values were found within the criteria limits, and it demonstrated good stability during the study period at all QC levels. Contrariwise results were observed for chlorogenic acid QC solutions that were unstable at period under consideration as depicted by RE (%) and the percentage difference between the derived concentrations at the two time points of analysis and under the particular storage condition (% change). Particularly, the measured concentration of chlorogenic acid was elevated by 37–57% for the three QC levels, and the differences were assigned statistically significant according to *p*-values (P<0.05) (Table S9). For the case of IS, a −12% (P<0.05) concentration reduction was observed post 1 month of storage. Considering the inadequate stability of chlorogenic acid and IS, and in order avoid possible counterfeits in our results, stock solutions of all standard compounds were prepared weekly, and working standard solutions as well as QCs were prepared daily. Moreover, sample solutions were analyzed within 3 days post preparation.

Table S9. Summary of stability results for analytes and internal standard using the proposed LC- MS/MS method.

			Short term (Bench top stability, 4 h)			Long term (1 month)				
**Compound**	**QC sample**	**Nominal concentration (μg·mL^{-1})**	**Recovered concentration (μg·mL^{-1})[1]**	**RSD[1] (%) (N=3)[2]**	**RE[3] (%)**	**Recovered concentration (μg·mL^{-1})[1]**	**RSD (%) (N=3)**	**RE (%)**	**% Change**	***P*-value**
Chlorogenic acid	LQC	0.60	0.612(±0.025)[4]	4.1	2.0	0.838(±0.035)	4.2	40	37	0.0008
	MQC	1.2	1.236(±0.076)	6.2	3.0	1.945(±0.015)	0.78	62	57	0.0001
	HQC	2.0	2.058(±0.069)	3.3	2.9	3.214(±0.087)	2.7	61	56	0.0001
Naringenin	LQC	0.012	0.01300(±0.00060)	4.6	8.3	0.0125(±0.0014)	11.0	4.4	-3.6	0.5660
	MQC	0.024	0.0260(±0.0018)	7.0	8.3	0.0258(±0.0019)	7.5	7.5	-0.80	0.8980
	HQC	0.040	0.0408(±0.0023)	5.6	2.0	0.0363(±0.0018)	5.0	-9.4	-11	0.0546
4-Chloro-4′-hydroxybenzophenone (IS)		1.0	1.012(±0.053)	5.2	1.2	0.8906(±0.0033)	0.37	-11.0	-12	0.0487

[1] RSD: relative standard deviation; [2] N: Number of QC replicates; [3] RE: relative error,; [4] Values are presented as mean(±standard deviation).

References

1. EMA. European Medicines Agency, Guideline of Bioanalytical Method Validation. Available online: https://www.ema.europa.eu/en/documents/scientific-guideline/guideline-bioanalytical-method-validation_en.pdf (accessed on 7 September 2021).
2. FDA. US Food and Drug Administration, Bioanalytical Method Validation—Guidance for Industry. Available online: https://www.fda.gov/files/drugs/published/Bioanalytical-Method-Validation-Guidance-for-Industry.pdf (accessed on 7 December 2021).
3. ICH. International Council for Harmonisation of Technical Requirements for Pharmaceuticals for Human Use, ICH Harmonised Guidelines M10—Bioanalytical Method Validation (Draft Version). Available online: https://www.ema.europa.eu/en/documents/scientific-guideline/draft-ich-guideline-m10-bioanalytical-method-validation-step-2b_en.pdf (accessed on 7 September 2021).
4. Cittan, M.; Çelik, A. Development and Validation of an Analytical Methodology Based on Liquid Chromatography–Electrospray Tandem Mass Spectrometry for the Simultaneous Determination of Phenolic Compounds in Olive Leaf Extract. *J. Chromatogr. Sci.* **2018**, *56*, 336–343. https://doi.org/10.1093/chromsci/bmy003.
5. Liu, Z.; Tu, M.-J.; Zhang, C.; Jilek, J.L.; Zhang, Q.-Y.; Yu, A.-M. A Reliable LC-MS/MS Method for the Quantification of Natural Amino Acids in Mouse Plasma: Method Validation and Application to a Study on Amino Acid Dynamics during Hepatocellular Carcinoma Progression. *J. Chromatogr. B* **2019**, *1124*, 72–81. https://doi.org/10.1016/j.jchromb.2019.05.039.
6. Faccin, H.; Viana, C.; Nascimento, P.C.D.; Bohrer, D.; de Carvalho, L.M. Study of ion suppression for phenolic compounds in medicinal plant extracts using liquid chromatography–electrospray tandem mass spectrometry. *J. Chromatogr. A* **2016**, *1427*, 111–124. https://doi.org/10.1016/j.chroma.2015.12.017.
